# Genomic and plasmid profiles of multidrug-resistant *Salmonella* isolates from pediatric patients from Zhuhai, China

**DOI:** 10.1128/spectrum.04063-25

**Published:** 2026-04-21

**Authors:** Yongru Chen, Yanmei Fang, Kaisong Huang, Huitao Huang, Lejia Zhao, Xinfeng Ji, Yixin Sun, Xuemei Yang, Jiubiao Guo, Zhiming Cai

**Affiliations:** 1 Clinical Research Center, The First Affiliated Hospital of Shantou University Medical College, Shantou, China; 2 Zhuhai Center for Disease Control and Prevention, Zhuhai, China.; 3 Guangdong Provincial Key Laboratory of Medical Immunology and Molecular Diagnostics, Guangdong Medical University, Dongguan, China; 4 The State Key Laboratory of Pharmaceutical Biotechnology, School of Life Sciences, Nanjing University, Nanjing, Jiangsu, China; 5 Department of Laboratory Medicine, Nanjing Drum Tower Hospital, Nanjing University Medical School, Nanjing, Jiangsu, China; 6 Department of Pharmacology, Shantou University Medical College, Shantou, China; 7 Shenzhen University Carson International Cancer Center, Shenzhen, China; Institute for Biomedical Research on Retroviruses and AIDS (INBIRS), Buenos Aires, Argentina

**Keywords:** *Salmonella*, multidrug-resistant, pediatric patients, *tra *gene, plasmid transferability

## Abstract

Multidrug-resistant *Salmonella* has been classified as a pathogen of high priority. However, limited or mixed-quality studies reported multidrug-resistant *Salmonella* strains from pediatric patients. The study aimed to investigate the prevalence, serotypes, antimicrobial characteristics, and plasmid profiles of clinical *Salmonella* strains isolated from pediatric patients. For that, we collected 46 *Salmonella* isolates from children’s fecal samples in multiple hospitals. Antimicrobial susceptibility testing, conjugation assay, short-read and long-read sequencing, and bioinformatics analysis were performed. Our data revealed that the top three serotypes of the 46 isolated strains are *Salmonella* I 4,[5],12:i:-, Typhimurium, and Enteritidis, with prevalence at 34.78% (16/46), 23.91% (11/46), and 17.39% (8/46), respectively. Most of these strains clustered into ST34, ST19, and ST11. Antimicrobial susceptibility testing analysis suggested that more than 80% of these *Salmonella* isolates belong to multidrug-resistant strains, all of them showed resistance to ampicillin, ampicillin-sulbactam, and trimethoprim-sulfamethoxazole, and all strains were susceptible to meropenem, with about 17.39% of them having minimum inhibitory concentrations ≥4 µg/mL to imipenem. Moreover, conjugation assay and long-read sequencing analysis on the plasmids harbored in 12 of the 46 *Salmonella* strains consistently showed that none of them has intact conjugative transfer genes, indicating *Salmonella* strains from pediatric patients have different antimicrobial resistance levels and impaired plasmid transferability due to loss of part or whole *tra* genes.

**IMPORTANCE:**

*Salmonella*, one of the world’s top prevalent foodborne pathogens, has gained resistance to fluoroquinolone and many other drugs that are used clinically. However, few studies focused on the genomic and plasmid profiles of multidrug-resistant *Salmonella* isolates from pediatric patients. In the present report, our data indicate that *Salmonella* strains isolated from pediatric patients have different antimicrobial resistance levels and plasmid transferability compared to those from adults. These findings provide crucial insights into pediatric patients’ isolated *Salmonella* strains from Zhuhai, China, which can be leveraged to optimize drug regimens, leading to more effective and less toxic treatments for pediatric infections, and call for more attention to *Salmonella* epidemiology and antimicrobial resistance patterns in children.

## INTRODUCTION

*Salmonella* is a leading cause of foodborne illness, hospitalizations, and deaths globally, and children under 5 years old are one of the groups of people who have a higher infection risk (1, 2). In China, pediatric patients face distinct challenges in the management of *Salmonella* infections, particularly due to the high prevalence and severity of the disease in young children. Surveillance data from Guangzhou, China (2018–2023) indicate that the overall positive rate of *Salmonella* isolated from stool samples was 22.0%, with *Typhimurium* as the predominant serotype of *Salmonella* isolates (63.1%, 823/1,304) (3). Treatment choices have traditionally been third-generation cephalosporins; however, recent data reveal that sensitivity to these frontline agents has declined dramatically (2, 4). Multidrug-resistant (MDR) *Salmonella* that is resistant to at least one agent in three or more antimicrobial classes, especially the increasing emergence of fluoroquinolone-resistant *Salmonella* species, has been classified as a pathogen of high priority (5). The emergence of MDR *Salmonella* poses an even greater therapeutic challenge, with MDR rates reaching 48.5% in some pediatric cohorts (4). The challenge is compounded by significant differences between pediatric and adult populations. In children, non-typhoid Salmonella bacteremia was usually secondary to gastroenteritis, whereas in most adults, non-typhoid Salmonella infection presented as a primary bacteremia (6). Notably, while children with non-typhoid Salmonella bacteremia exhibit a higher MDR rate compared to adults (42.9% vs 16.7%) (7). These findings underscore the urgent need for further studies on the unique epidemiological and resistance patterns of *Salmonella* infections in children.

Antimicrobial resistance (AMR) among *Salmonella* could be acquired by mutations in chromosomal DNA, and horizontal gene transfer mechanisms allowing for rapid, multidirectional, and cross-species sharing of resistance genes is another more important strategy for the acquisition and spread of AMR genes (8, 9). Conjugative plasmids encoding transfer genes (*tra*) are required for conjugation, which is a primary route for AMR gene transfer (10, 11). However, limited or mixed quality studies reported the status of *tra* genes and transferability of plasmids in multidrug-resistant *Salmonella* strains isolated from pediatric patients. Zerrin Aktas et al. characterized 41 pediatric isolates of Salmonella spp. collected in Turkey, found that plasmids from resistant strains were not transferred by conjugation (12). With an aim to provide more information on *Salmonella* AMR profiles and conjugation capability in children, and to guide anti-infection therapy for pediatric patients, the present study analyzed the features of plasmids in *Salmonella* from children under the age of five, and the genomic profiles of multidrug-resistant *Salmonella* isolates were analyzed as well.

## MATERIALS AND METHODS

### Bacterial isolates and identification

Fecal samples following the inclusion criteria that pediatric patients under the age of five, with fever and diarrhea and culture confirmed as *Salmonella* infection, from four hospitals (two baby-friendly hospitals and two grade A tertiary hospitals) in Zhuhai, China, were collected in 2019. Verbal informed consent for samples collection and subsequent analysis was obtained from caregivers of enrolled patients. *Salmonella* isolates were recovered as previously described with minor modifications (13, 14). Briefly, sample homogenates were inoculated into LB broth and incubated at 35°C for 24 h for enrichment. One milliliter aliquot of pre-enriched homogenate was transferred to 9 mL of Tetrathionate broth (Difco) and incubated at 42°C for another 24 h, then streaked on XLT4 agar for purification twice. The matrix-assisted laser desorption ionization time-of-flight mass spectrometry biotyper system (Bruker, Germany) was utilized to confirm the species identity of all strains.

### Antimicrobial susceptibility testing

By following a previous study (14), antimicrobial susceptibility profiles were determined using broth microdilution or agar dilution method according to the guidelines of the Clinical and Laboratory Standards Institute. Minimum inhibitory concentrations (MICs) were assessed for 11 antibiotics: ampicillin (AMP), ampicillin-sulbactam (SAM), ceftazidime (CAZ), cefepime (FEP), meropenem (MEM), imipenem (IPM), minocycline (MIN), ciprofloxacin (CIP), levofloxacin (LVX), chloramphenicol (CHL), and trimethoprim/sulfamethoxazole (SXT). *Escherichia coli* ATCC 25922 was used as the quality control strain.

### Whole genome, plasmid sequencing, and bioinformatics analysis

Genomic DNA was extracted using the PureLink Genomic DNA Mini Kit (Invitrogen, USA). Short-read sequencing libraries were prepared using the DNBseq PE150 platform (2 × 150 bp paired-end), while long-read sequencing was performed using the Oxford Nanopore MinION platform (Oxford Nanopore Technologies, UK). Integrated quality control and sequencing data preprocessing were performed by using the SOAPnuke software (15). The long reads were assembled using Canu v1.5 (16).

Assembled genome sequences were annotated with RAST v2.0 (17). Detection of the multilocus sequence typing (MLST) was determined by MLST 2.0 (18). The molecular serovar was identified by SISTR (19). The core phylogenetic tree of the 46 strains was built with IQ-tree according to the Panaroo pipeline (20, 21). Plasmid typing was determined by Plasmid MLST (22). Alignment of plasmids was generated by Easyfig_win_2.1 (23).

### Conjugation assay

To assess the horizontal transferability of the plasmids sequenced in different *Salmonella* strains, either streptomycin-resistant *E. coli* C600 or sodium azide-resistant J53 strains was used as the recipient, and corresponding antibiotics selection strategies were applied based on the AMR profiles of strains. Other experimental procedures were performed by following previous description (14). Briefly, donor and recipient strains were separately cultured, mixed at a 1:4 (vol/vol) ratio, and filter mating method was used. After statistical incubation at 37°C for 24 h, transconjugants were selected on agar plates containing corresponding antibiotics depending on donor and recipient strains tested. All experiments were performed in triplicate.

### Nucleotide sequence accession numbers

The whole-genome sequences of the 34 short-read sequenced isolates and the 12 long-read sequenced isolates have been deposited in GenBank under the accession numbers SAMN53769895–SAMN53769929 and SAMN53770111–SAMN53770121.

## RESULTS

### Characteristics of the 46 isolated *Salmonella* strains

Based on the inclusion criteria, feces from patients under the age of five with fever and diarrhea were retrospectively enrolled. *Salmonella* isolates defined as MDR were selected for further analysis. As detailed in Table 1, for the enrolled patients, 20 out of 46 (43.48%) were aged under 1, 25 out of 46 (54.35%) were aged between 1 and 4 years old, and 1 (2.17%) neonate was included. The top three serotypes of the 46 isolated strains are *Salmonella* I 4,[5],12:i:-, Typhimurium, and Enteritidis, with prevalence at 34.78% (16/46), 23.91% (11/46), and 17.39% (8/46), respectively.

**TABLE 1:** Characteristics of the 46 isolated *Salmonella* strains from pediatric patients

Strain^*a*^	Age^*b*^	Serotype	ST type
008	3 yr	Typhimurium	ST19
011	9 mo	Enteritidis	ST11
013	10 mo	Typhimurium	ST19
015	1 yr	I 4,[5],12:i:-	ST34
018	3 yr	I 4,[5],12:i:-	ST29
041	10 mo	Typhimurium	ST19
048	8 mo	Typhimurium	ST19
054	1 yr and 5 mo	Enteritidis	ST11
056	2 yr	Typhimurium	ST34
059	8 mo	Stanley	ST19
060	1 yr and 9 mo	Enteritidis	ST11
064	1 yr	I 4,[5],12:i:-	ST34
066	8 mo	I 4,[5],12:i:-	ST34
067	1 yr and 3 mo	Typhimurium	ST19
073	1 yr and 6 mo	I 4,[5],12:i:-	ST34
074	2 yr	Agona	ST13
085	Neonate	Typhimurium	ST34
089	2 yr	Enteritidis	ST11
091	2 mo	I 4,[5],12:i:-	ST34
092	2 yr	Typhimurium	ST19
094	9 mo	I 4,[5],12:i:-	ST34
095	4 yr	Typhimurium	ST34
099	3 yr	I 4,[5],12:i:-	ST34
175	8 mo	I 4,[5],12:i:-	ST34
187	6 mo	Muenster	ST321
193	2 yr	Typhimurium	ST19
197	2 yr and 1 mo	Othmarschen	ST23
201	1 yr and 3 mo	I 4,[5],12:i:-	ST34
206	1 yr and 6 mo	Enteritidis	ST11
207	11 mo	Derby	ST40
214	1 yr and 4 mo	I 4,[5],12:i:-	ST34
225	8 mo	I 4,[5],12:i:-	ST34
232	8 mo	I 4,[5],12:i:-	ST34
233	2 yr	Typhimurium	ST19
236	1 yr and 1 mo	Enteritidis	ST11
238	1 yr	London	ST155
244	9 mo	I 4,[5],12:i:-	ST34
245	10 mo	Enteritidis	ST11
246	1 yr	I 4,[5],12:i:-	ST34
249	2 yr	Albany	ST292
250	1 yr and 1 mo	Saintpaul	ST27
251	1 yr and 9 mo	I 4,[5],12:i:-	ST34
254	2 yr	Enteritidis	ST11
258	1 yr and 11 mo	Paratyphi B	ST43
292	2 yr	Infantis	ST32
297	10 mo	Kentucky	ST198

^*a*^ All strains were isolated from fecal samples.

^*b*^ yr stands for year, and mo stands for month.

### AMR profiles and phylogenetic analysis of sequenced *Salmonella* isolates

Antimicrobial susceptibility testing results (Table 2) showed more than 80% of these strains belong to MDR. All the 46 *Salmonella* isolates resistant to ampicillin, ampicillin-sulbactam, and trimethoprim-sulfamethoxazole, with MICs ≥64 µg/mL, ≥32/16 μg/mL, and ≥4.75/152 μg/mL, respectively. About 80.43% (37/46) isolates were resistant to chloramphenicol (MICs ≥ 32 µg/mL), 26 out of the 46 (56.52%) strains displayed resistance to minocycline, 32.61% (15/46) and 30.43% (14/46) were resistant to ceftazidime and cefepime, respectively. Moreover, 26.09% (12/46) of these *Salmonella* isolates were resistant to ciprofloxacin, the levofloxacin resistance rate was slightly higher than that to ciprofloxacin (28.26%, 13/46), eight isolates (17.39%) had MICs ≥4 µg/mL to imipenem. Additionally, all the strains were susceptible to meropenem, with MICs ranging from 0.06 to 0.5 μg/mL.

**TABLE 2:** Minimum inhibitory concentrations of the 46 isolated *Salmonella* strains^*a*^

	Minimum inhibitory concentration (μg/mL)										
Strain	AMP	SAM	CAZ	FEP	MEM	IPM	MIN	CIP	LVX	CHL	SXT
008	64	32	0.5	0.25	0.06	0.25	16	0.5	1	64	152
011	64	32	0.5	0.25	0.06	0.25	4	0.25	0.5	4	9.5
013	64	64	0.5	0.25	0.06	0.25	16	0.5	1	64	152
015	64	16	0.5	1	0.06	0.25	16	2	2	64	152
018	64	16	4	1	0.06	0.25	32	2	2	64	152
041	64	64	16	16	0.06	0.5	16	0.5	1	64	152
048	64	32	2	16	0.06	0.25	4	0.5	1	64	9.5
054	64	32	0.5	0.5	0.06	0.25	16	0.25	1	8	19
056	64	16	0.5	1	0.06	0.25	8	2	2	64	152
059	64	32	4	0.5	0.06	0.25	≤1	0.5	0.125	64	9.5
060	64	32	0.5	0.5	0.06	0.25	4	0.03	0.125	8	152
064	64	32	0.5	2	0.125	4	16	2	2	64	19
066	64	64	4	8	0.125	1	4	0.5	1	64	152
067	64	32	0.5	1	0.125	4	4	0.25	1	64	152
073	64	64	16	4	0.5	4	16	0.5	0.5	64	152
074	64	32	0.5	1	0.25	8	8	0.25	1	64	9.5
085	64	16	0.5	1	0.25	0.25	16	2	2	64	152
089	64	32	0.5	1	0.06	4	8	0.125	0.5	4	9.5
091	64	16	0.5	0.5	0.06	0.25	16	0.03	0.125	64	4.75
092	64	16	16	16	0.06	0.25	4	0.5	1	4	152
094	64	64	16	16	0.06	0.5	32	0.5	2	64	152
095	64	32	0.5	2	0.06	4	16	2	2	64	152
099	64	16	16	16	0.06	0.5	16	0.25	0.5	8	4.75
175	64	32	16	16	0.06	0.5	32	0.25	1	32	9.5
187	64	32	16	16	0.06	0.25	16	0.5	4	64	152
193	64	16	0.5	0.25	0.06	0.5	16	0.25	1	64	152
197	64	32	0.5	0.25	0.06	0.5	16	0.5	1	64	152
201	64	32	16	2	0.06	0.25	32	0.5	1	64	152
206	64	64	1	0.25	0.06	2	4	0.25	0.5	8	9.5
207	64	32	2	2	0.06	0.25	8	2	2	64	152
214	64	64	0.5	0.25	0.06	0.25	4	0.5	1	64	152
225	64	32	16	16	0.25	0.25	32	2	4	64	152
232	64	32	1	1	0.06	0.25	8	4	4	64	152
233	64	32	1	4	0.06	0.25	4	0.5	0.5	64	152
236	64	32	16	1	0.06	0.25	8	8	4	64	152
238	64	16	16	16	0.06	0.25	4	0.03	0.125	64	4.75
244	64	32	1	4	0.06	0.25	4	0.25	0.25	64	152
245	64	64	8	16	0.06	0.25	16	0.5	1	64	152
246	64	32	16	16	0.06	0.5	16	0.5	1	64	152
249	64	64	2	0.5	0.06	8	16	0.25	1	64	152
250	64	32	16	16	0.06	0.25	16	1	4	64	152
251	64	64	8	16	0.06	0.25	16	0.5	1	64	152
254	64	64	0.5	0.5	0.06	4	2	0.25	0.5	8	9.5
258	64	64	16	16	0.06	0.25	2	0.06	0.125	16	4.75
292	64	32	2	1	0.06	0.25	16	1	1	64	152
297	64	64	16	0.25	0.125	0.5	16	0.5	1	64	152

^*a*^ AMP, ampicillin; SAM, ampicillin-sulbactam; CAZ, ceftazidime; FEP, cefepime; MEM, meropenem; IPM, imipenem; MIN, minocycline; CIP, ciprofloxacin; LVX, levofloxacin; CHL, chloramphenicol; and SXT, trimethoprim/sulfamethoxazole.

**Fig 1:**
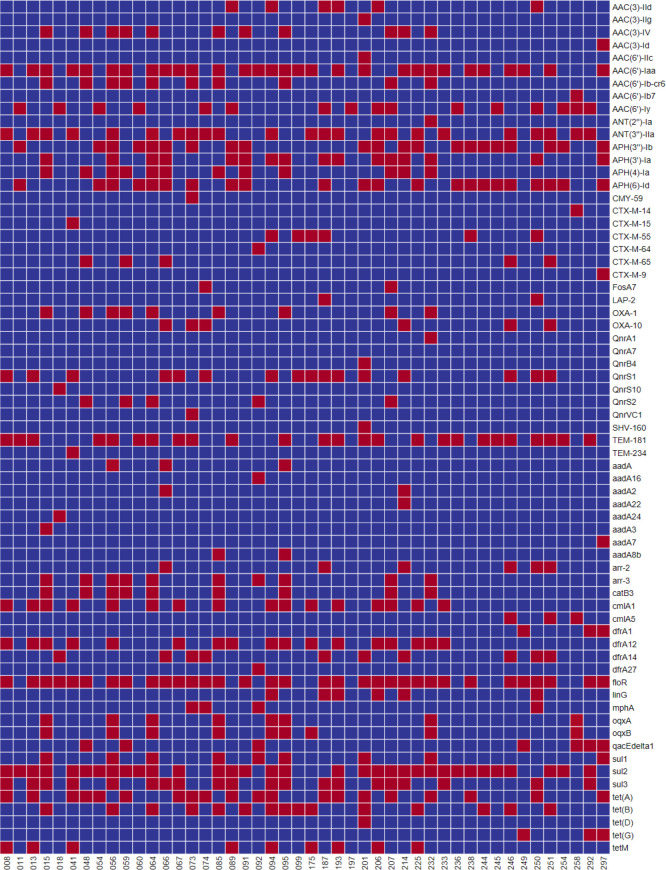
Antimicrobial resistance gene profiles of the 46 *Salmonella* strains. Red and blue indicated the presence and absence of the corresponding antimicrobial resistance genes, respectively.

Whole-genome sequencing data analysis on resistance genes in different strains (Fig. 1) indicated that among all the 46 strains, ampicillin resistance in more than 50% was mediated by TEM-181, TEM-234, CTX, SHV-160, and OXA, which contributed to ampicillin resistance in some other strains. And some isolates harbor more than one type of ampicillin-resistant gene. Sul1, sul2, and sul3 were identified in the strains resistant to trimethoprim-sulfamethoxazole, with sul2 as the most common resistance gene. Resistance to chloramphenicol was associated with *cmlA1* and/or *floR*. The widespread aminoglycoside antibiotic resistance genes aadA, aph, aac, and ant were present in most of the 46 isolated *Salmonella* strains.

**Fig 2:**
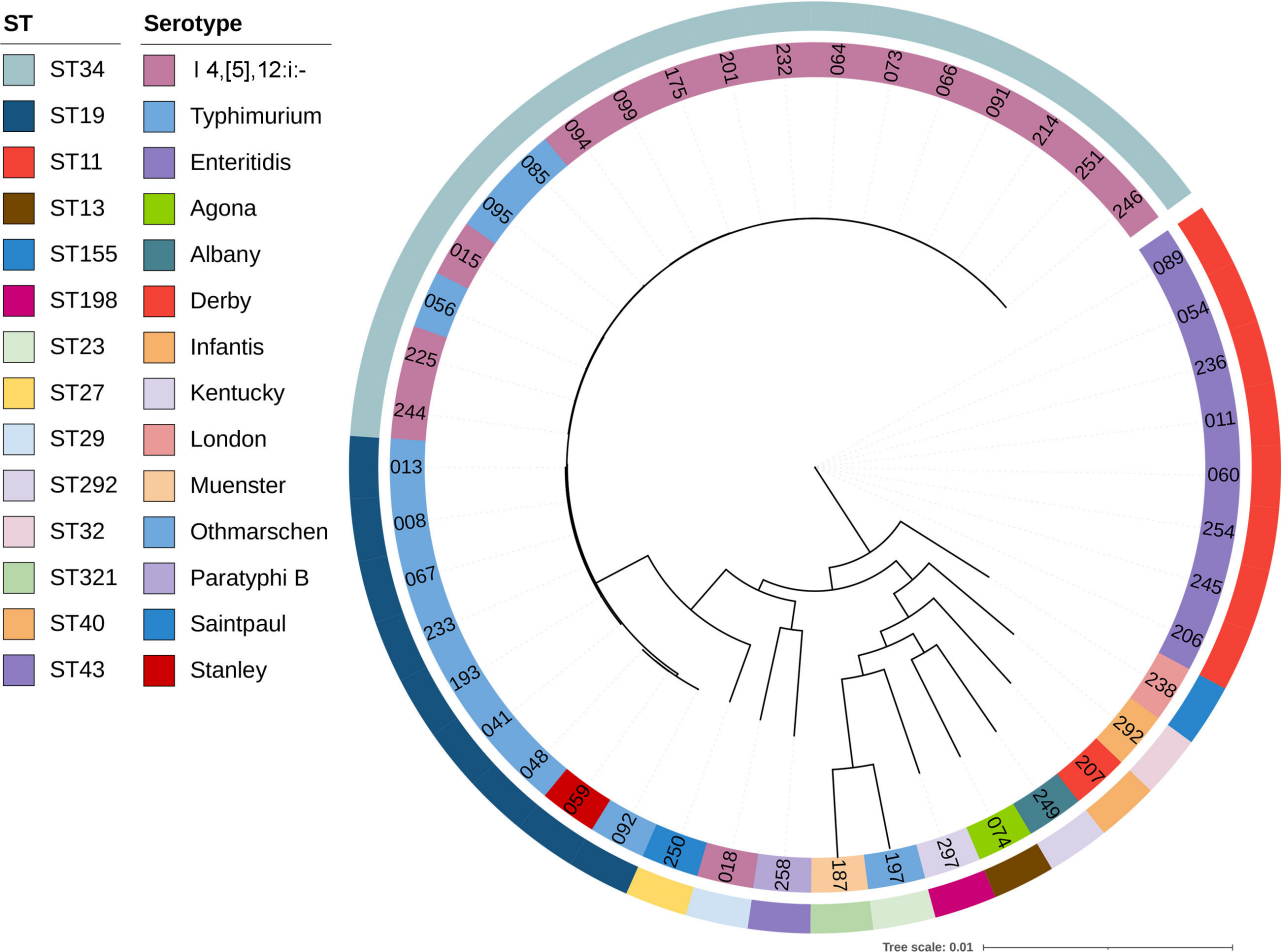
Phylogenetic tree of the 46 *Salmonella* strains. The inner circle represents serotype, and the outer circle represents ST type.

The phylogenetic tree showed that the 46 strains clustered into four major groups (Fig. 2). Eighteen ST34 strains comprising three Typhimurium and 16 of its monophasic variants I 4,[5],12:i:- formed a tight cluster with minimal genetic distances. The remaining Typhimurium strains belonged to ST19 and formed a distinct cluster separate from ST34 isolates. Interestingly, the Stanley serotype strain 059 was placed within the ST19 cluster, indicating close genetic relatedness. *Salmonella* Stanley and *Salmonella* Typhimurium share the same O-antigen and phase-2 flagellar antigen, differing only in the phase-1 flagellar antigen. ST19 is a well-established clonal complex known to underlie multiple serotypes, and divergence in the flagellar loci can give rise to different serotypes within the same core-genome lineage. The eight ST11 Enteritidis strains also grouped together. These three STs account for about 65% of all isolates, representing the core locally transmitted clones. The remaining strains each represented a unique ST type and serotype, suggesting independent introduction events rather than local transmission chains.

### Plasmids' genetic characteristics in different *Salmonella* strains

**Fig 3:**
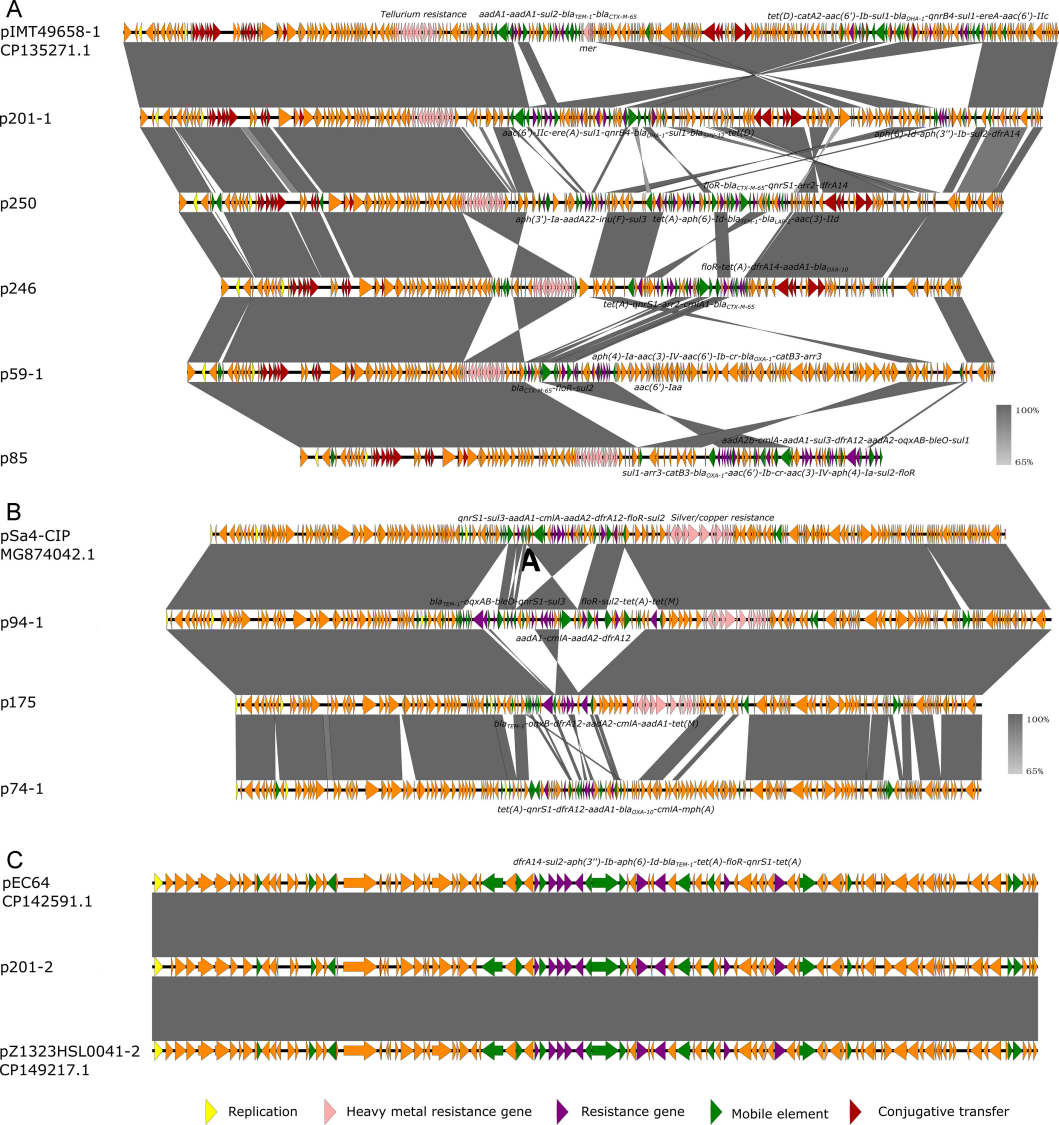
Characterization of the MDR plasmids carried by the *Salmonella* isolates. (**A**) Alignment of the IncHI2 plasmids with similar plasmids. (**B**) Alignment of the IncFIA/HI1 plasmids with similar plasmids. (**C**) Alignment of plasmid p201-2 with similar plasmids.

To further characterize the plasmid profiles in the *Salmonella strains*, 12 isolates representing the range of serotypes, STs, and resistance profiles observed in the full collection were selected for long-read sequencing (Table S1). Ten of these strains were found to carry at least one plasmid, whereas no plasmid was identified in strains 018 and 187. Notably, although plasmids were detected in 207 and 238, these plasmids did not carry any resistance genes. In all four of these isolates, resistance genes were located exclusively on the chromosome. A total of nine MDR plasmids were identified from the remaining eight strains (Table 3). The five IncHI2 plasmids shared a similar backbone and carried a diverse MDR region encoding genes conferring resistance to aminoglycosides, beta-lactams, fluoroquinolones, macrolides, phenicols, rifampicin, sulfamethoxazole, tetracycline, and trimethoprim (Table 3; Fig. 3A). Among these, plasmid p201-1 showed the highest similarity (93% coverage and 100% identity) to a plasmid from *Enterobacter hormaechei* strain IMT49658-1 (24) (Table 3; Fig. 3A). This provides strong evidence for horizontal transfer of IncHI2 plasmids across different genera of Enterobacteriaceae, suggesting that these plasmids serve as a shared reservoir of resistance genes across pathogenic and commensal bacteria, which might facilitate the spread of AMR within clinical and community settings. IncHI2 plasmids are frequently reported in *Salmonella* and have played a key role in the emergence and dissemination of multidrug resistance (25). Notably, the IncHI2 plasmids identified here carried only partial conjugative transfer genes, which likely explains their observed non-transferability (26), consistent with our conjugation assay results. Three IncFIA/HI1 plasmids were identified among these strains (Table 3; Fig. 3B). Two of these, p94-1 and p175, originated from serotype I 4,[5],12:i:- isolates and shared 100% sequence identity, although p175 lacked a portion of the mosaic multidrug resistance region. The third plasmid, p74-1 (from a serotype Agona strain), was missing the genomic region encoding silver and copper resistance compared to plasmids p175 and p94-1. Blast against the NCBI database, plasmids p94-1 exhibited highest similarity (93% coverage and 99.99% identity) to plasmid pSa4-CIP from *Salmonella* sp. strain Sa4 (Fig. 3B). In addition to the IncHI2 plasmid, a smaller plasmid (93,372 bp) was identified in strain 201 (Table 3; Fig. 3C). This plasmid, designated p201-2, showed 100% coverage and 99.9% identity to a plasmid from *Escherichia coli* strain 64 and to plasmid pZ1323HSL0041-2 from *Salmonella enterica* subsp. enterica serovar I 4,[5],12:i:- strain Z1323HSL0041 (Fig. 3C). None of these related plasmids carried intact conjugative transfer genes. These results indicate that this non-conjugative MDR plasmid has disseminated across different genera of Enterobacteriaceae, and the presence of nearly identical plasmids in both *Salmonella* and *E. coli* underscores the role of commensal bacteria as a reservoir for resistance genes that can be acquired by pathogens.

**TABLE 3:** Characterization of the MDR plasmids carried by the *Salmonella* isolates

Plasmid	Size (bp)	Inc type	Resistance genes	*tra* gene
p059-1	254,212	HI2	*aph(4)-Ia, aac(3)-IV, aac(6')-Ib-cr, aac(6')-Iaa, bla*_CTX-M-65_ *, bla*_OXA-1_ *, floR, catB3, arr-3, sul2*	Partial
p074-1	222,266	FIA/HI1	*aadA1, bla*_OXA-10_ *, mph(A), cml, qnrS1, tet(A), dfrA14*	–^*a*^
p085	183,242	HI2	*aph(4)-Ia, aadA1, aadA2, aadA2b, aac(3)-IV, aac(6')-Ib-cr, bla*_OXA-1_ *, cmlA1, floR, catB3, oqxAB, arr-3, sul1, sul2, sul3, dfrA12, bleO*	Partial
p094-1	263,990	FIA/HI1	*aadA1, aadA2, bla*_TEM-1_*, cmlA, floR, oqxAB, qnrS1, sul2, sul3, tet*(M)*, tet*(A)*, dfrA14, bleO*	–
p175	222,583	FIA/HI1	*bla*_TEM-1_*, oqxB, dfrA12, aadA2, cmlA1, aadA1, tet*(M)	–
p201-1	284,091	HI2	*aph(3'')-Ib, aph(6)-Id, aac(6′)-IIc, bla*_DHA-1_*, bla*_SHV-12_*, ere(A), qnrB4, sul1, sul2, tet*(D)*, dfrA14*	Partial
p201-2	93,372	/^*b*^	*aph(3'')-Ib, aph(6)-Id, bla*_TEM-1_*, floR, qnrS1, sul2, tet*(A)*, dfrA14*	–
p246	233,062	HI2	*aadA1, bla*_CTX-M-65_*, bla*_TEM-1_*, bla*_OXA-10_*, cmlA1, floR, qnrS1, arr-2, tet*(A)*, dfrA14*	Partial
p250	259,524	HI2	*aac(3)-IId, aph(3′)-Ia, aph(6)-Id, aadA22, bla*_CTX-M-55_*, bla*_TEM-1_*, bla*_LAP-2_*, mph*(A)*, lnu*(F)*, floR, qnrS1, arr-2, sul3, tet*(A)*, dfrA14*	Partial

^*a*^ “–” indicates that *tra* gene is absent in the corresponding plasmid.

^*b*^ “/” indicates that no known Inc group in the corresponding plasmid.

## DISCUSSION

International surveillance networks and most studies on multidrug-resistant organisms are focusing on adult populations. Several reports showed that microorganism carriage rate and antibiotic resistance patterns were different in children (2, 27). *Salmonella*, one of the world’s top prevalent foodborne pathogens, has gained resistance to multidrug, and the infection by MDR *Salmonella* strains has led to rising morbidity, mortality, and economic costs globally (5, 28). A detailed study on genomic and plasmid profiles of multidrug-resistant *Salmonella* isolates from pediatric patients is essential for pediatric antimicrobial stewardship and useful to guide pediatric patients' anti-infection therapy.

The present study showed that *Salmonella* I 4,[5],12:i:-, Typhimurium, and Enteritidis were the top three serotypes, with prevalence at 34.78% (16/46), 23.91% (11/46), and 17.39% (8/46), respectively, with a total prevalence at about 76.09% (35/46), and most of these strains clustered into ST34, ST19, and ST11. A higher isolation rate of these serotypes from different populations was reported. Qiongdan Mai et al. found in Guangzhou, China, the isolate rate of *Salmonella* Typhimurium and Enteritidis serotypes from children was 65.2% (3). Moreover, Salmonella I 4,[5],12:i:-, Typhimurium, and Enteritidis serotypes isolated rate from children was 66.13%, compared with that of 52.56% from adults in Hangzhou, China (29). In addition, for the clinical characteristics of non-typhoidal Salmonella bacteremia, respiratory diseases and acute enteritis were the top two most common comorbidities accounted for in children; however, cerebrovascular disease and malignancy were significantly more prevalent in adults (7). These studies suggest continuous surveillance of Salmonella epidemiology, drug resistance, and clinical characteristics in children is of great significance to guide anti-infection therapy in Chinese children, as well as worldwide pediatric patients. Antimicrobial susceptibility testing analysis suggested that more than 80% of these *Salmonella* isolates belong to MDR strains; all of them showed resistance to ampicillin, ampicillin-sulbactam, and trimethoprim-sulfamethoxazole. The high ampicillin-resistant rate found in the present study is consistent with that reported by the Infectious Disease Surveillance of Pediatrics program (2), but is higher than the global AMR level (30, 31). Moreover, all the strains included in our study were susceptible to meropenem, and about 17.39% of them had MICs ≥4 µg/mL to imipenem, much lower than the data of AMR rates in *Salmonella* isolates in China (28). Due to the relatively limited sample size in the present study, a more conclusive conclusion may be difficult to reach now, but a glimpse of the difference in *Salmonella* AMR levels from pediatric patients could be caught.

Conjugation is a major driver of bacterial genomes' rapid evolution, facilitating bacterial strains' adaptation of resistance to antimicrobials (11). *Tra* region in plasmid clustered with transfer genes plays pivotal roles in conjugation, the expression of these *tra* genes initiates the conjugation process, forms T4SS and conjugative pilus prior to transfer (32). Previous studies reported successful conjugation results from *Salmonella* isolates (13, 25); however, few articles have focused on the transferability of plasmids isolated from pediatric patients' *Salmonella* strains. In our study, all conjugation assays failed, and long-read sequencing data analysis showed that none of these related plasmids carried intact conjugative transfer genes. These findings suggest that multidrug-resistant *Salmonella* isolates may be circulating within the local environment. Furthermore, the genetic relatedness of plasmids across different strains raises the possibility of mother-to-child transmission as one route through which pediatric patients may acquire MDR *Salmonella*. The result was not anticipated. One possible explanation for the conjugative transfer genes lost in these plasmids is that the mother-to-child transmission route may play a role in determining and affecting the conjugation capability of plasmids from *Salmonella* isolates in pediatric patients. Another possibility is that other factors, not only plasmid intrinsic properties that are responsible for conjugation capability, bacteria density and metabolites, host autacoid or exogenous compounds in intestinal microecological environments, may function in plasmid horizontal gene transfer (33, 34). But, due to limited samples in the present study and lack of direct experimental evidence from available literature, it is difficult to approach a conclusive view currently. Further studies, involving more samples from children of various ages and comparing plasmid composition and conjugation efficiency of identical *Salmonella* serotypes isolated from both children and adults, are necessary to uncover the potential factors and underlying mechanisms influencing the loss of conjugative transfer genes or plasmid conjugation capacity in pediatric patients.

In conclusion, we reported and analyzed the genomic and plasmid profiles of multidrug-resistant *Salmonella* isolates from pediatric patients. The difference in AMR levels and plasmid transferability displayed by these strains requires more attention to *Salmonella* and other pathogenic bacteria as well, epidemiology, and resistance in children. Though consistent findings regarding *Salmonella* prevalent serotypes and MDR rate from children were reported in the present study and some other investigations, findings, particularly regarding mother-to-child transmission route and the loss of conjugative transfer genes in *Salmonella,* should be viewed as hypothesis-generating rather than conclusive. It is crucial to acknowledge the constraints imposed by geographic regionality and sample size in the present work, multi-center with larger and balanced cohorts surveillance of antimicrobial resistance of *Salmonella* isolated from pediatric patients, comparison with that of adults, and laboratory results are of great significance for a better understanding of *Salmonella* epidemiology in children, and further effectively fight against *Salmonella* infection in pediatric patients.

## Data Availability

The data sets presented in this study are available in online repository. The name of the repository and accession numbers are provided in the article.

## SUPPLEMENTAL MATERIAL

Table S1 spectrum.04063-25-s0001.docx The long-read sequencing assembly results of the selected 12 *Salmonella* strains.

ASM does not own the copyrights to Supplemental Material that may be linked to, or accessed through, an article. The authors have granted ASM a non-exclusive, world-wide license to publish the Supplemental Material files. Please contact the corresponding author directly for reuse.
