# Supplementary material for: Genomic and plasmid profiles of multidrug-resistant Salmonella isolates from pediatric patients from Zhuhai, China
Source: Microbiol Spectr. 2026 Apr 21;14(6):e04063-25. doi: 10.1128/spectrum.04063-25 (PMC13228062; doi:10.1128/spectrum.04063-25)
Supplement: Table S1 — The long-read sequencing assembly results of the selected 12 Salmonella strains. [file spectrum.04063-25-s0001.docx]

**Genomic and** **plasmid profiles of multidrug-resistant *Salmonella* isolates from pediatric patients from Zhuhai, China**

Yongru Chen^1#^, Yanmei Fang^2#^, Kaisong Huang^3^, Huitao Huang^2^, Lejia Zhao^4,5^, Xinfeng Ji^1^, Yixin Sun^6^, Xuemei Yang^4,5*^, Jiubiao Guo^1,6*^, Zhiming Cai^1,7*^

**^1^** Clinical Research Center, The First Afﬁliated Hospital of Shantou University Medical College, Shantou, China.

**^2^** Zhuhai Center for Disease Control and Prevention, Zhuhai, China.

**^3^** Guangdong Provincial Key Laboratory of Medical Immunology and Molecular Diagnostics, Guangdong Medical University, Dongguan, China.

**^4^** The State Key Laboratory of Pharmaceutical Biotechnology, School of Life Sciences, Nanjing University, Nanjing, Jiangsu, China.

**^5^** Department of Laboratory Medicine, Nanjing Drum Tower Hospital, Nanjing University Medical School, Nanjing, Jiangsu, China.

**^6^** Department of Pharmacology, Shantou University Medical College, Shantou, China.

**^7^** Shenzhen University Carson International Cancer Center, Shenzhen, China.

**^#^ Yongru Chen and Yanmei Fang contributed equally to this article. Author order was determined alphabetically.**

**^*^ Correspondence**: Zhiming Cai, caizhiming2000@163.com; Jiubiao Guo, jbguo@stu.edu.cn; Xuemei Yang, xuemei.yang@nju.edu.cn.

**Table S1** The long-read sequencing assembly results of the selected 12 *Salmonella* strains.

| **Strain ID** | **Sequence ID** | **Topology** | **Total Length (bp)** | **GC Content (%)** |
| --- | --- | --- | --- | --- |
| 018 | Chromosome | circular | 4,810,543 | 52.23 |
| 059 | Chromosome | circular | 4,809,240 | 52.21 |
|  | Plasmid1 | circular | 254,212 | 47.95 |
|  | Plasmid2 | circular | 93,859 | 53.10 |
| 074 | Chromosome | circular | 5,021,575 | 51.91 |
|  | Plasmid1 | circular | 222,266 | 47.54 |
|  | Plasmid2 | circular | 92,109 | 46.93 |
|  | Plasmid3 | circular | 48,524 | 44.26 |
| 085 | Chromosome | circular | 4,896,880 | 52.17 |
|  | Plasmid | circular | 183,242 | 46.91 |
| 094 | Chromosome | circular | 5,029,974 | 52.16 |
|  | Plasmid1 | circular | 263,990 | 47.74 |
|  | Plasmid2 | circular | 36,861 | 52.78 |
| 175 | Chromosome | circular | 4,938,857 | 52.18 |
|  | Plasmid1 | circular | 222,583 | 46.55 |
| 187 | Chromosome | circular | 4,782,945 | 52.16 |
| 201 | Chromosome | circular | 4,997,850 | 52.19 |
|  | Plasmid1 | circular | 284,091 | 46.85 |
|  | Plasmid2 | circular | 93,372 | 50.32 |
| 207 | Chromosome | circular | 4,884,809 | 52.09 |
|  | Plasmid | circular | 70,231 | 47.77 |
| 238 | Chromosome | circular | 4,785,343 | 52.19 |
|  | Plasmid | circular | 93,948 | 47.52 |
| 246 | Chromosome | circular | 5,059,239 | 52.14 |
|  | Plasmid | circular | 233,062 | 46.25 |
| 250 | Chromosome | circular | 4,829,627 | 52.19 |
|  | Plasmid | circular | 259,524 | 46.74 |
